# The Panoramic ECAP Method: Estimating Patient-Specific Patterns of Current Spread and Neural Health in Cochlear Implant Users

**DOI:** 10.1007/s10162-021-00795-2

**Published:** 2021-04-23

**Authors:** Charlotte Garcia, Tobias Goehring, Stefano Cosentino, Richard E. Turner, John M. Deeks, Tim Brochier, Taren Rughooputh, Manohar Bance, Robert P. Carlyon

**Affiliations:** 1 Medical Research Council Cognition & Brain Sciences Unit, University of Cambridge, 15 Chaucer Road, Cambridge, CB2 7EF UK; 2 Department of Engineering, University of Cambridge, Trumpington Street, Cambridge, CB2 1PZ UK; 3 Cambridge Universities Hospital Foundation Trust, University of Cambridge, Hills Road, Cambridge, CB2 0QQ UK; 4 Cambridge Hearing Group, Cambridge, UK

**Keywords:** cochlear implant (CI), electrically evoked compound action potential (ECAP), neural excitation patterns, neural health, current spread, optimization

## Abstract

The knowledge of patient-specific neural excitation patterns from cochlear implants (CIs) can provide important information for optimizing efficacy and improving speech perception outcomes. The Panoramic ECAP (‘PECAP’) method (Cosentino et al. 2015) uses forward-masked electrically evoked compound action-potentials (ECAPs) to estimate neural activation patterns of CI stimulation. The algorithm requires ECAPs be measured for all combinations of probe and masker electrodes, exploiting the fact that ECAP amplitudes reflect the overlapping excitatory areas of both probes *and* maskers. Here we present an improved version of the PECAP algorithm that imposes biologically realistic constraints on the solution, that, unlike the previous version, produces detailed estimates of neural activation patterns by modelling current spread and neural health along the intracochlear electrode array and is capable of identifying multiple regions of poor neural health. The algorithm was evaluated for reliability and accuracy in three ways: (1) computer-simulated current-spread and neural-health scenarios, (2) comparisons to psychophysical correlates of neural health and electrode-modiolus distances in human CI users, and (3) detection of simulated neural ‘dead’ regions (using forward masking) in human CI users. The PECAP algorithm reliably estimated the computer-simulated scenarios. A moderate but significant negative correlation between focused thresholds and the algorithm’s neural-health estimates was found, consistent with previous literature. It also correctly identified simulated ‘dead’ regions in all seven CI users evaluated. The revised PECAP algorithm provides an estimate of neural excitation patterns in CIs that could be used to inform and optimize CI stimulation strategies for individual patients in clinical settings.

## INTRODUCTION

Although many cochlear-implant (CI) listeners understand speech well in quiet backgrounds, there is much variability in outcomes, particularly in noisy conditions (Friesen et al. 2001; Firszt et al. 2004). Knowledge about each CI user’s unique neural activation pattern can provide important information for optimizing efficacy and improving speech perception (Long et al. 2014; Pfingst et al. 2015). Here we describe a method for using the electrically evoked compound action-potential (ECAP) (Charlet de Sauvage et al. 1983; Brown et al. 1990) to provide an objective measurement of two key factors affecting neural activation patterns, namely the current spread from each electrode and the pattern of neural health along the cochlea. Several studies have suggested that ECAPs can be used to estimate aspects of neural activation patterns. For example, experiments with guinea pigs have shown that spiral-ganglion neuron (SGN) survival is positively correlated with the maximum ECAP amplitude, the IPG effect on the ECAP amplitude, and with the slope of the ECAP Amplitude Growth Function (AGF) (Prado-Guitierrez et al. 2006; Ramekers et al. 2014). The IPG effect is the impact on the ECAP metric of increasing the gap between the two phases of a biphasic current pulse (i.e., from 8 to 58 µs), giving the stimulated neurons more time to recover from their response to the first phase when presented with the second phase (Prado-Guitierrez et al. 2006).

**Fig. 1:**
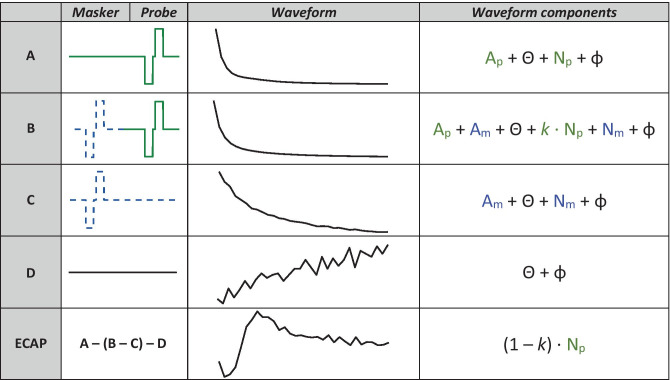
Schematics and components for the forward-masking artefact-reduction technique. The maskers (dashed blue lines) and probes (solid green lines) are presented in biphasic current units, and the waveforms (solid black lines) were measured in C28 (with the probe and masker both on electrode 3) in µV (*y* axis) over a period of 2 ms (*x* axis). *A*_*p*_ is the stimulus artefact as a result of the probe, *A*_*m*_ is the same for the masker, *N*_*p*_ is the neural response to the probe, *N*_*m*_ is the same for the masker, Θ is the amplifier switch-on artefact, *φ* is baseline neural activity, and *k* is the proportion of the probe’s neural response that is not masked by the masker. For maskers and probes presented on the same electrode, the entire neural response to the probe is masked. Therefore, *k* = 0 and the resulting ECAP waveform is *N*_*p*_

A widely used method to estimate neural spread of excitation (SOE) using ECAPs was first presented by Cohen et al. (2003) and leverages the Forward-Masking method of artefact reduction (Abbas et al. 1999). As CI stimulation artefacts are much larger than the evoked neural responses, artefact-reduction techniques are necessary to extract the neural response. In the Forward-Masking method, the responses to several combinations of probe and masker pulses are recorded and a subtraction paradigm is used to extract the neural response to the probe pulse (Fig. 1). This method requires the neural response to the probe pulse to be completely masked by the masker pulse, and the reduction in ECAP amplitude with increasing masker-probe distance has been considered an estimate of the neural SOE from the probe (Abbas et al. 2004; Hughes and Abbas 2006; van der Beek et al. 2012). A few metrics derived from these data have been shown to correlate with estimates of channel interaction within the cochlea. The equivalent rectangular bandwidth (ERB) of the area under the ECAP SOE function has been shown to correlate with electrode-modiolus distances measured from CT scans (DeVries et al. 2016), which are commonly thought to affect current spread. Another metric, the Channel Separation Index (CSI), is calculated by taking two SOE functions centred on different electrodes and summing the difference between their respective normalized ECAP amplitudes as a function of masker location (Hughes 2008). This produces a higher CSI for electrodes which produce less similar SOE functions and has been shown to be a significant predictor of speech perception scores (Scheperle and Abbas 2015). However, both of the above SOE and CSI metrics are oversimplifications, because the amplitude of the ECAP waveform is determined by the spread from both the probe and the masker electrodes. Essentially, these methods assume uniform (and, in fact, infinitely narrow) spatial spread from each masker electrode, which is likely not the case.

To overcome this limitation, Cosentino et al. (2015) introduced the Panoramic ECAP Method (also referred to simply as ‘PECAP’), which considers the variation of the neural activation patterns by collecting ECAP amplitudes for all combinations of masker and probe electrodes. Based on a multi-stage nonlinear optimization algorithm, PECAP correctly identified notable exceptions to ideal electrode-neuron interfaces such as cross-turn stimulation and computer-simulated areas of neural death. However, it was limited in its detection to a single exception per CI user (i.e. one neural dead region or instance of cross-turn stimulation), and the constraints imposed on the algorithm—which were necessary for it to reach a unique solution—were not biologically plausible. For example, the algorithm did not allow for the asymmetric neural excitation patterns that are likely to occur in the event of the presence of a neural dead region. Another method has also been presented using similar data and treating it as a deconvolution problem (Biesheuvel et al. 2016).

Here we present a revised Panoramic ECAP Method that also uses ECAP amplitudes for all combinations of masker and probe electrodes. It primarily differs from the original PECAP method in that it explicitly models the data using a combination of assumed current spread from each electrode and a common patttern of estimated neural health along the length of the cochlea. We argue that this allows the model to account for effects such as neural dead regions and broad current spread using a set of biologically realistic constraints; other differences from the original method are described in the following section.

We evaluated the revised PECAP method in three ways. First, we performed computer simulations to generate patterns of neural health and current spread, simulated the ECAPs that these would produce, added noise to those measures, and submitted this artificial ECAP matrix to the algorithm. This allowed us to compare the PECAP outputs to a ‘ground truth’ and to evaluate its robustness to noise. It also provided an illustration of some of the algorithm’s potential benefits and limitations. It was, however, somewhat circular in design and therefore limited in terms of its ecological validity. Next we used some previous data including ECAP recordings, focused detection thresholds, and CT scans in human CI users to test the hypotheses that regions of lower predicted neural health according to the PECAP algorithm should correspond to regions with higher detection thresholds obtained with focussed stimulation and that wider current spread estimations according to the PECAP algorithm should correspond to electrodes that are located further away from neural tissue. Finally, we recorded ECAPs from a group of CI users and manipulated the stimuli so as to simulate the effects of a neural dead region. The resulting data were then submitted to the PECAP algorithm, allowing us to determine whether it could successfully identify the locations of the simulated neural dead regions in the cochlea.

## METHODS: DATA COLLECTION

### Participants

Data were analysed from two cohorts of CI users, one of which was collected in a previous study and the other in the present study. The data from the first cohort were generously provided by DeVries et al. (2016), who collected ECAP amplitudes for all available combinations of probes and maskers using the Forward-Masking artefact-reduction technique, presented at most comfortable level (MCL). These ECAP data were available for 9 of the 10 participants included in that study, all of whom were users of Advanced Bionics devices. ECAPs from a tenth participant were only available for a few electrodes and were therefore excluded from our analysis. Steered quadrupolar (sQP) thresholds and electrode-modiolus distances calculated from computerized tomography (CT) scans were also available in the same participants. (For details, see DeVries et al. 2016.)

The second cohort was all volunteers at the MRC Cognition and Brain Sciences Unit in Cambridge, UK. Permission to conduct the study was granted by the National Research Ethics committee for the East of England, and all participants provided their written consent to participate. They were reimbursed for their travel costs and were compensated for volunteering their time. Thirteen users of Cochlear Corporation devices were recruited; three of whom were excluded from the analysis either because no ECAPs could be measured or because stimulation artefacts obscured the majority of responses. Those retained in the analysis are described in Table 1. All participants were unilaterally implanted with the exception of C19, who was sequentially bilaterally implanted and for whom both ears were tested (the two ears are referred to as C19R and C19L). This resulted in inclusion of a total of 11 ears with participants at an average of 62.2 years of age (standard deviation = 14.6 years). Electrodes were excluded from PECAP recording either if they were deactivated in the clinical MAP, or if they elicited non-auditory sensations.

**Table 1:** Participant information

ID	Implanted ear	Age (years)	Duration of profound hearing loss before implantation (years)	Duration of implant use (years)	Aetiology	Device	Electrodes assessed	ECAP recording gain (dB)/delay (µs)	Dead region simulation centre (electrode)
C03	Right	75	10	15	Scarlet fever, viral infections, and Meniere’s disease	CI24RE	4–22	50 dB/122 µs	16
C04	Left	75	52 (L) 15 (R)	14	Idiopathic viral infection	CI24RE	4–22	40 dB/122 µs	n/a
C09	Right	69	48	14	Hereditary	CI24RE	2–21	40 dB/98 µs	15
C13	Right	57	17	4	Maternal rubella and ear infection	CI522	1–22	50 dB/122 µs	16
C19R	Right	66	18	4	Exposure to loud sounds	CI422	3–22	50 dB/98 µs	5
C19L	Left	66	20	2	Exposure to loud sounds	CI512	1–20	50 dB/122 µs	10
C25	Left	43	14	2.5	Hereditary	CI522	4–22	50 dB/122 µs	n/a
C26	Right	57	3	2	Hereditary	CI522	1–22	60 dB/122 µs	n/a
C28	Right	30	14	12	Perinatal deafness due to ototoxic antibiotic	Hybrid L24	4–22	40 dB/73 µs	8
C29	Left	76	gradual decline	3	Exposure to loud sounds	CI522	3–22	50 dB/98 µs	n/a
C30	Right	71	5	4	Measles	CI512	1–22	60 dB/73 µs	14

*n/a* not applicable

### Panoramic Electrically Evoked Compound Action-Potential Measurements

The data collection methods described in this section refer only to the second cohort of participants described above and included in Table 1. Participants were asked to remove their clinical CI processor and replace it with a laboratory-owned CP910 processor connected to a testing laptop computer running the Custom Sound EP clinical software (Cochlear Limited, Australia). If participants wore a hearing device in their contralateral ear, they removed it during the session. We initially performed some preliminary measurements so as to identify the stimulus levels and recording parameters that would be used in the main part of the study. For these preliminary measures, ECAPs were obtained using the forward-masking technique in the “Advanced NRT” tab of the Cochlear Corporation clinical software program, Custom Sound EP. Symmetric, cathodic-leading biphasic current pulses with phase durations (PD) of 25 µs, inter-phase gaps (IPGs) of 8 µs, masker offsets of 0 Current Units (CUs), and masker-probe intervals (MPIs) of 400 µs that were presented at 80 pulses per second (pps) on one electrode at a time, starting from subthreshold current levels. All stimuli were presented in monopolar (MP) mode, with the extra-cochlear ball electrode (MP1) used as the ground for stimulation and the case electrode (MP2) used as the ground for recording. Participants were instructed to report loudness using an eleven-point chart with descriptors labelled from 0 (inaudible) to 10 (too loud). Current levels were increased in steps of 6, 4, or 2 current units (CUs) until participants reported a loudness level of 7 (loud but comfortable), and then decreased in steps of 2 CUs until the participant indicated that loudness was at level 6 (MCL). The CUs at the perceptual detection threshold, as well as for loudness levels of 4 (medium soft), 5 (medium), 6, and 7, were recorded. If the current level reached the upper compliance limit for an electrode prior to reaching the participant’s perception of loudness level 7, then the PD (for every electrode) was increased to 42 µs (this occurred for participants C04, C09, and C28). This procedure was performed for every third electrode that was turned on in the participant’s clinical MAP. For electrodes for which loudness scaling was not performed, the CUs for MCL were interpolated linearly between evaluated electrodes.

Using an electrode that had been observed to have a clear ECAP waveform during loudness scaling, recording parameters for the Gain and Delay were optimized for each participant using the ‘Optimize recording parameters’ option in Custom Sound EP. Delays from 73 to 122 µs in step sizes of 25 µs as well as Gains of 40, 50, 60, and 70 dB at a recording electrode offset of +2 were evaluated. While the Delay and Gain settings should not affect ECAP amplitudes in theory, they may affect measurements by clipping the N1 peak, obscuring the waveform via amplifier saturation, or causing excessive measurement noise. Therefore, the Delay and Gain combination that produced the highest measured N1-P2 ECAP amplitude was selected, except in cases where the waveform that produced the highest amplitude was observed to be particularly noisy compared to average, in which case a Delay and Gain setting combination that produced a lower amplitude was selected in favour of a less noisy waveform. These parameters were then used for all subsequent recordings on all electrodes for that participant, the values for which are included in Table 1.

Custom software written in Python (version 2.4.4, Python Software Foundation, US) through the PyCharm IDE interface (Community Edition 2016.1.5, JetBrains, Czech Republic) was then used for recording ECAPs based on the NIC2 platform from Cochlear Corporation. The software allowed for the acquisition of four ECAP waveforms, each averaging 12 sweeps for a total of 48 sweeps per ECAP recording condition; accessible in the same amount of time, it would take to record a single 48-sweep waveform using Custom Sound EP. The collection of four individual average waveforms instead of a single one provided an estimate of the variability of the ECAPs and was used in the analysis of robustness of PECAP to noise. Stimulus parameters were the same as for the preliminary measurements.

**Fig. 2:**
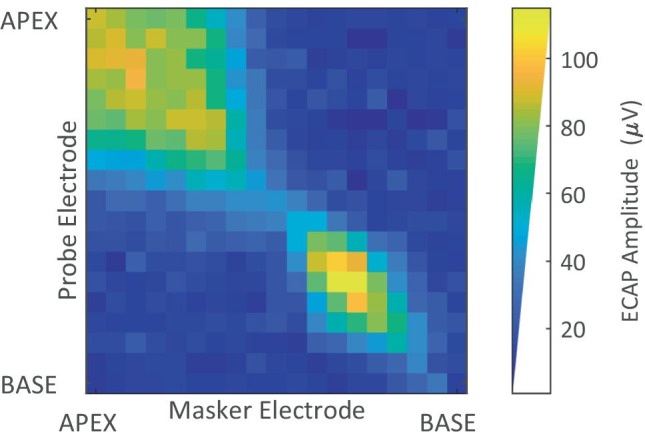
An example of the PECAP ${{\varvec{M}}}_{{\varvec{o}}}$ matrix measured for a CI participant (C03), each cell of which represents the amplitude of an ECAP waveform in µV, recorded using all possible combinations of probe and masker electrodes from base to apex. It can be seen that the ECAP amplitudes are highest along the diagonal of the matrix for which probe and masker are placed on the same electrode, thereby maximising the overlap in neural excitation area

The parameters for Gain, Delay, Phase Duration, IPG, and MCL current levels were used to record ECAP amplitudes for every electrode included in the participant’s active MAP (additionally excluding electrodes for which non-auditory sensations were reported) using the Forward-Masking artefact-reduction technique for the cases where the probe and the masker were co-located (on the same electrode). This subset of ECAP recordings will hereafter be referred to as the ‘diagonal’ as they constitute the diagonal of the recorded PECAP matrix (an example of which is shown in Fig. 2). Note that the current levels for both the masker and the probe were set to MCL and that the default 10-CU increase in the level of the masker relative to the probe that is used in the Custom Sound EP software was removed. ECAPs were recorded using an electrode spaced two electrodes apical from the active probe electrode, except on the two most apical electrodes where the recording electrode was placed two electrodes basal to the active probe electrode. It was confirmed with the participant that the stimulation level used for the diagonal was comfortable to listen to for an extended period of time prior to initiating the primary PECAP recording sequence, which consisted of recording an ECAP waveform for every combination of masker and probe electrode. The recording electrode was again located two electrodes apical to the probe electrode as a default during this primary recording sequence, with the recording electrode two basal to the probe for the two most-apical probes. When the recording electrode would have been co-located with the masker electrode, the recording electrode was moved to the electrode between the masker and the probe. These recordings constitute the primary PECAP matrix (hereafter referred to as ${{\varvec{M}}}_{{\varvec{o}}}$, and shown in Fig. 2). The PECAP recording sequence took between 35 and 55 min to record, depending on how many electrodes were deactivated in the participant’s MAP or excluded from the PECAP matrix due to non-auditory sensations. Participants were instructed to sit in a chair but were allowed to sleep, read, write, or watch documentaries with subtitles during the recording sequence.

### Dead Region Simulations

**Fig. 3:**
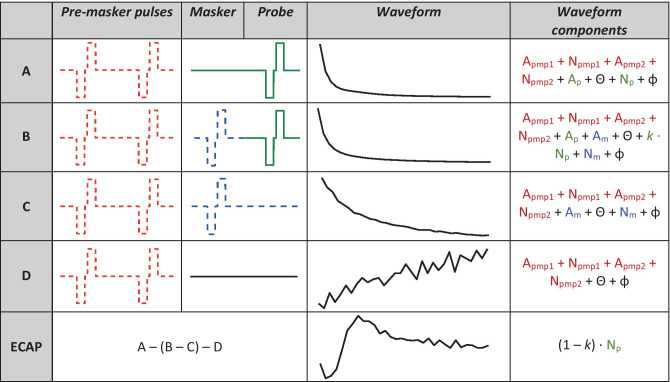
Schematics and components for the forward-masking artefact-reduction technique in the dead region simulation condition. Note that the onset of each of the biphasic current pulses (pre-masker pulses, maskers, and probes alike) was spaced 400 µs apart from each other. The additional symbols in the ‘waveform components’ column not described in Fig. 1 are in red and are as follows: *A*_pmp1_ is the stimulus artefact as a result of the first pre-masker pulse, *A*_pmp2_ is the same for the second pre-masker pulse, *N*_pmp1_ is the neural response to the first pre-masker pulse, and *N*_pmp2_ is the same for the second pre-masker pulse. With the addition of the two pre-masker pulses (red dotted lines) prior to the Masker and Probe pulses in the standard forward-masking artefact-reduction technique, there is no change to the result of the ECAP waveform equation, as the additional components are all subtracted out

For seven of the participants listed in Table 1, we collected a second ${{\varvec{M}}}_{{\varvec{o}}}$ matrix that included a neural dead region simulation. The Forward-Masking artefact-reduction technique requires four recorded frames to extract the neural response from the stimulus artefacts: the probe alone, the probe preceded by the masker, the masker alone, and a system signature (see Fig. 1). In order to simulate a neural dead region, one electrode was selected per participant on which two pre-masker pulses, spaced 400 µs apart from each other, were presented 400 µs before each of these frames for every masker-probe combination in the ${{\varvec{M}}}_{{\varvec{o}}}$ matrix (see Fig. 3). These pre-masker pulses were always presented on the same electrode, regardless of the masker-probe pair. It was reasoned that the nerves excited by the electrode selected to simulate the neural dead region at MCL would still be in a refractory state with lower responsiveness during every ECAP recorded in ${{\varvec{M}}}_{{\varvec{o}}}$, thereby simulating a neural dead region (or at least a region of reduced responsiveness) centred on one electrode. The aim was to determine whether PECAP would correctly identify this local reduction in neural responsiveness compared to the standard condition without the neural dead region simulation.

For participants for whom the dead region simulation dataset (${{\varvec{M}}}_{{\varvec{D}}{\varvec{R}}{\varvec{S}}}$) was collected during a different session than the standard ${{\varvec{M}}}_{{\varvec{o}}}$ dataset, loudness scaling as described in the previous session was repeated, to determine whether any changes in perceptual loudness for a given current level had occurred between sessions. This was not observed in any of the participants, so the same current levels were used in both sessions. The electrode used for the dead region simulation was selected by visually inspecting the ECAP amplitudes along the diagonal of ${{\varvec{M}}}_{{\varvec{o}}}$ in the standard condition and selecting an electrode with an ECAP amplitude considered high enough such that the expected reduction in response amplitude due to the pre-masker pulses would be detectable between the two conditions. The current level of the pre-masker pulses was set to the same level determined as MCL for that electrode during loudness scaling.

The diagonal of the PECAP matrix with the addition of the dead region simulation was then recorded using the custom NIC2 software, after which the participant was again asked whether the loudness level was comfortable to listen to for an extended period of time. For two participants, this was not the case. To decrease listening levels to a lower, more comfortable loudness, the level of the pre-masker pulses was decreased for C03 by 5 CUs while the masker and probe pulses remained at MCL. Participant C09, however, indicated that decreasing the pre-masker pulses by 5 CUs was still not comfortable for extended listening. In order to achieve comfortable listening levels for C09, both the ${{\varvec{M}}}_{{\varvec{o}}}$ and ${{\varvec{M}}}_{{\varvec{D}}{\varvec{R}}{\varvec{S}}}$ were additionally recorded at a loudness level of 4 (medium soft) on the loudness chart. The other five participants indicated that the diagonal with the dead region simulation was louder than for the standard PECAP diagonal, but that it was still comfortable to listen to for an extended period of time. Therefore, all current levels for these participants remained at the levels identified as MCL during initial loudness scaling. The electrodes selected for the dead region simulation condition for each participant are listed in Table 1.

## METHODS: PECAP ALGORITHM STRUCTURE

### Overview

This subsection provides an overview of the PECAP algorithm. More-detailed information including mathematical formulations are provided in the following subsection.

The PECAP algorithm assumes that each measured ECAP—that is, each cell of the measurement matrix ${\varvec{M}}$—is determined by the overlap of the excitation patterns produced by the corresponding masker and probe. To formalize this, we assume that there is another matrix, ${\varvec{A}}$, each row of which represents the neural excitation pattern produced by stimulating one electrode. We also assume that each excitation pattern results from the combination of two factors. One of these is given by a third matrix, ${\varvec{C}}$, each row of which represents the current spread produced by stimulating one electrode. We assume that each current-spread function is Gaussian and that the functions for each electrode differ only in the width (σ) of the Gaussian. The second factor is a neural health vector, *ƞ*, which varies along the electrode array. Its value is constrained to vary between 0 and 1, where 0 corresponds to a completely unresponsive—or dead—neural region. Note that because the input (${\varvec{M}}$) to the algorithm consists of ECAPs, it is deaf to non-synchronized responses and so *ƞ* predominantly reflects the synchronized neural responsiveness, and ${\varvec{A}}$ reflects the synchronized neural excitation patterns. Nevertheless, we use the terms ‘neural health’ (referring not directly to SGN survival but synchronized neural responsiveness measured with forward-masked ECAPs) and ‘excitation pattern’ for brevity.

**Fig. 4:**
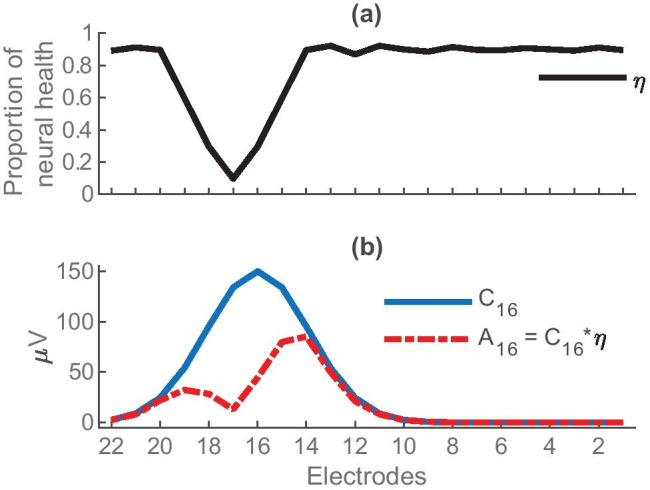
**a** An example of a neural health vector *ƞ* along the cochlea from apex (left) to base (right) with a dead region centred on electrode 17. **b** The Gaussian current spread (solid blue line) centred on electrode 16 (C_16_) and the resultant neural activation pattern (dashed red line) for the same electrode that is defined as *A*_16_ = *C*_16_ $\cdot$ *ƞ*

An example of how the current spread and neural health are combined to produce an excitation pattern is illustrated in Fig. 4a, which shows the neural-health vector (*ƞ*) as a function of position along the cochlea in the case of a neural dead region centred on electrode 17. The solid blue line in Fig. 4b shows the Gaussian current spread produced by stimulating electrode 16 (the 16th row of the ${\varvec{C}}$ matrix). Multiplying these two curves gives the predicted excitation pattern (the 16th row of the ${\varvec{A}}$ matrix) shown by the dashed red line Fig. 4b. It’s worth noting that although the current spread is constrained to be symmetric wthin ${\varvec{C}}$, the algorithm can reproduce an asymmmetric excitation pattern in ${\varvec{A}}$. In addition, the neural-health vector *ƞ*, which is independent of which electrodes are being stimulated, will have a biolgically plausible and consistent effect on excitation patterns produced by stimulating other nearby electrodes. For example, given the neural health *ƞ* in Fig. 4a, stimulating electrode 15 would also produce an asymmetric excitation pattern with less activation near electrode 16 than electrode 14.

**Fig. 5:**
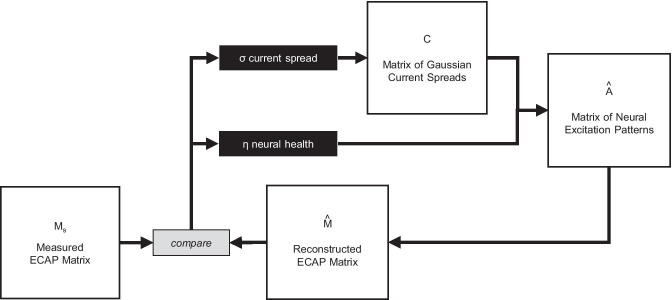
Schematic for the PECAP algorithm. The optimization algorithm adjusts the values in the *σ* and *ƞ* vectors, reconstructs $\widehat{M}$, and updates *σ* and *ƞ* iteratively in order to minimize the RMSE between ${M}_{o}$ and $\widehat{M}$

An overview of the PECAP estimation process is shown in Fig. 5. The input to the algorithm is the measurement matrix ${\varvec{M}}$. The algorithm generates some initial random values for the current-spread matrix ${\varvec{C}}$ and of the neural-health vector *ƞ*. Next, the algorithm combines *ƞ* and ${\varvec{C}}$ to produce the predicted excitation patterns, and then calculates the ECAP matrix that would arise from those predicted excitation patterns. This predicted ${\varvec{M}}$ matrix ($\widehat{{\varvec{M}}}$) is then compared to the observed ${\varvec{M}}$ matrix (${{\varvec{M}}}_{{\varvec{o}}}$), and the initial estimates of ${\varvec{C}}$ and *ƞ* are updated using a nonlinear optimization algorithm. This procedure continues iteratively in such a way as to minimize the error between ${{\varvec{M}}}_{{\varvec{o}}}$ and $\widehat{{\varvec{M}}}$ until a stop criterion is met, at which point the algorithm returns the final estimates of *σ* and *ƞ*, together with the resulting estimates of the excitation patterns (${\varvec{A}}$).

Before describing the mathematical details of the PECAP, it is worth making an important general point, known as the inverse problem (Groetsch 1993; Kirsch 1996). This is that, although a given set of excitation patterns will, according to the model, produce a unique predicted measurement matrix ($\widehat{{\varvec{M}}}$), the converse is not true. That is, a given measurement matrix ${{\varvec{M}}}_{{\varvec{o}}}$ could in theory arise from any one of a large number of underlying excitation patterns (${\varvec{A}}$). This is why it is necessary to impose smoothness and boundary constraints on *ƞ* and ${\varvec{C}}$ (described in detail in the following section) in order to arrive at a stable and plausible solution. These essentially prevent the estimated parameters (*σ* and *ƞ*) from varying erratically between adjacent electrodes.

### Mathematical Formulation and Details

The following subsection provides the details of the mathematical formulas that govern the structure and implementation of the PECAP algorithm.

The primary qualitative advantage of the revised PECAP method over previous approaches is its ability to separately model the contributions of current spread and neural health. It estimates how both entities vary along the length of the electrode array, and allows for asymmetric patterns of neural excitation through their interaction. It is important to reiterate here that the neural health estimate does not directly represent SGN survival. As it is a parameter extrapolated from ECAP measurements, each cell of the neural-health vector described in this section represents the synchronous responsiveness of the auditory nerve at a given electrode relative to all other electrodes along the array for any given patient. The following section explains how the estimation of both the current spread and the neural health is achieved, given various assumptions about their interaction.

As previously noted, the PECAP model assumes that ECAP amplitudes are a result of the overlap in the neural activation patterns between the probe and masker electrodes. We can describe the amplitude of the ECAP from any given combination of masker and probe electrodes as

1 $${M}_{p,m}{=M}_{m,p}={\sum }_{k={e}_{1}}^{{e}_{n}}{A}_{p}\left(k\right)\cdot {A}_{m}\left(k\right)$$

where ${A}_{p}\left(k\right)$ and ${A}_{m}\left(k\right)$ are the neural activation patterns produced by electrodes $p$ (probe) and $m$ (masker) at MCL as a function of location along the cochlea $k$, expressed at each evaluated electrode in a CI user’s MAP from ${e}_{1}$ to ${e}_{n}$, where $n$ is the total number of active electrodes. We make the symmetry assumption ${M}_{p,m}{=M}_{m,p}$ because the overlapping area of neural excitation between two electrodes should be equivalent regardless of the order in which the probe and masker electrode pulses are presented. In reality, the measured ECAP amplitudes do not always exactly satisfy the ${M}_{p,m}{=M}_{m,p}$ assumption, due to a combination of measurement noise and the recording electrode being determined by the location of the probe electrode, rather than the masker electrode. Therefore, prior to submitting ${{\varvec{M}}}_{{\varvec{o}}}$ to the PECAP algorithm, each ECAP amplitude is replaced with an average amplitude, equivalent to averaging across the diagonal of ${{\varvec{M}}}_{{\varvec{o}}}$ (see Fig. 2), thereby also reducing the total amount of noise present in the data:

2 $${{M}^{^{\prime}}}_{p,m}{={M}^{^{\prime}}}_{m,p}= \frac{{M}_{p,m}+{M}_{m,p}}{2}$$

As noted above, each cell of this measurement matrix is assumed to arise from the overlap of the neural excitation patterns produced by the corresponding masker and probe. That is, if ${\varvec{A}}$ is the matrix of neural excitation patterns with the rows and columns corresponding to the excitation pattern of each probe and masker, respectively, then ${M}_{p,m}$ is the result of multiplying row $p$ and column $m$ of ${\varvec{A}}$. Because the excitation pattern produced by stimulating an electrode is the same whether it is used as masker or probe, the same is true if we transpose ${\varvec{A}}$ so that the rows represent the maskers and the columns represent the probes (${{\varvec{A}}}^{\mathbf{T}}$). Therefore, more generally, the full matrix containing the ECAPs for every combination of masker and probe electrode can be described as

3 $${\varvec{M}}=\sqrt{{\varvec{A}}\cdot {{\varvec{A}}}^{\mathbf{T}}}$$

where the cells in both the neural response (${\varvec{A}}$) and the ECAP (${\varvec{M}}$) matrices are expressed in units of in µV.

As described above and illustrated in Fig. 4, the PECAP algorithm allows for biologically plausible asymmetry of the neural excitation patterns by defining ${\varvec{A}}$ as the combination of two underlying factors: symmetric current spread from each electrode represented by a matrix ${\varvec{C}}$ for which each row is a Gaussian function, and a vector $\eta$ representing neural health at each electrode which is multiplied with each row of ${\varvec{C}}$ to obtain ${\varvec{A}}$:

4 $${\varvec{A}}=\eta \cdot {\varvec{C}}$$

Each row of ${\varvec{C}}$ is described by a Gaussian function, the shape of which is controlled by three parameters within the following equation:

5 $${C}_{i}\left(k\right)={\mathrm{\alpha }}_{i}\cdot {e}^{{-\frac{\left(k-{\upmu }_{i}\right)}{2\cdot {\upsigma }_{i}}}^{2}}$$

The amplitude ${\alpha }_{i}$, the mean ${\mu }_{i}$, and the standard deviation ${\sigma }_{i}$ of the Gaussian function describe the current spread from electrode $i$. By expressing ${\varvec{A}}$ as a linear combination of $\eta$ and ${\varvec{C}}$, we allow for modelling asymmetry in the neural activation patterns. Figure 4 shows an example of the neural activation pattern centred on one electrode for a situation where there is a neural dead region near the electrode in question (Fig. 4a), resulting in an asymmetric neural excitation pattern for that electrode (Fig. 4b). Note that the dead region will affect excitation patterns for all nearby electrodes in a biologically plausible and consistent manner, for example, affecting the apical side of the excitation patterns of more-basal electrodes, but the basal side of the excitation patterns of more-apical electrodes.

The PECAP algorithm assumes that the Gaussian current-spread from each electrode is centred on the $i$th electrode, so ${\mu }_{i}$ is also not a free parameter. To reduce free parameters and to enable both the ${\varvec{A}}$ and the $\mathbf{M}$ matrices to be in the same units of µV, all values of the amplitude variable $\alpha$ are set to a single, fixed value equal to the maximum ECAP amplitude of the ${{\varvec{M}}}_{o}$ matrix. Therefore, the only free parameter in the Gaussians in ${\varvec{C}}$ is $\sigma$, and Eq $\left(5\right)$ can be simplified to

6 $${C}_{i}\left(k\right)=\mathrm{\alpha }\cdot {\mathrm{e}}^{-\frac{{\left(k\right)}^{2}}{2\cdot {\upsigma }_{i}}}$$

Given the measured ${{\varvec{M}}}_{{\varvec{o}}}$ as an input to the PECAP algorithm, we wish to determine how the neural-health (${\eta }_{i}$) and current-spread (${\sigma }_{i}$) values vary along the length of a participant’s cochlea. As there is no unique solution for the two vectors $\eta$ and $\sigma$ that will produce any given ${{\varvec{M}}}_{{\varvec{o}}}$ due to the inverse problem mentioned above, a numerical approach using smoothness constraints and nonlinear optimization is used, as described below.

As depicted in Fig. 5, the optimization algorithm adjusts the neural-health and current-spread vectors $\eta$ and $\sigma$ iteratively to minimize the error between ${{\varvec{M}}}_{{\varvec{o}}}$ and $\widehat{{\varvec{M}}}$. For a set of electrodes 1: $N$, the estimation of neural health along the length of the cochlea $\eta$ is initialized with $N$ random values between 0 and 1 indicating a proportion of healthy neurons. The current spread parameter $\sigma$ is initialized with an additional $N$ random values between 1 and 6 indicating the standard deviation of the current spread in units of electrodes. The ${\varvec{C}}$ matrix is then defined using Eq. $\left(6\right)$ for $i$ = 1:$N$ for the randomly initialized values of $\sigma$. ${\varvec{A}}$ is defined using $\left(4\right)$, and $\widehat{{\varvec{M}}}$ is constructed using $\left(3\right)$. The root-mean-squared error (RMSE) between ${{\varvec{M}}}_{{\varvec{o}}}$ and $\widehat{{\varvec{M}}}$ is then calculated as

7 $${\varepsilon }_{{{\varvec{M}}}_{{\varvec{o}}},\widehat{{\varvec{M}}}}=\sqrt{\frac{1}{N}\cdot \sum {\left({{M}_{o}}_{p,m}-{\widehat{M}}_{p,m}\right)}^{2}}$$

and it is this value that is minimized during optimization.

The optimization method used for finding the values for $\eta$ and $\sigma$ with minimum ${\varepsilon }_{{{\varvec{M}}}_{{\varvec{o}}},\widehat{{\varvec{M}}}}$ is Sequential Quadratic Programming (sqp) implemented with the *fmincon()* function in MATLAB R2018a (Powell 1983; Hock and Schittowski 1983). PECAP uses inequality constrains to implement smoothness conditions that prevent adjacent electrodes from having values of $\sigma$ that vary by more than ± 3 for adjacent electrodes, and values of $\eta$ that vary by more than ± 0.3, which can be described as

8a $$\left|{\sigma }_{i}-{\sigma }_{i-1}\right|\le 3$$

8b $$\left|{\eta }_{i}-{\eta }_{i-1}\right|\le 0.3$$

as well as upper and lower limits consistent with $\sigma$ and $\eta$ initialization described as

9a $$0<{\eta }_{i}\le 1$$

9b $$1<{\sigma }_{i}\le 6$$

As previously noted, the neural-health vector $\eta$ does not directly represent absolute SGN survival and function. Therefore, ${\eta }_{i}$ = 0 does not mean there are no surviving SGNs at electrode $i$, nor does ${\eta }_{i}$ = 1 refer to a scenario where all SGNs are perfectly healthy at electrode $i$. The neural health (also described as the synchronized neural responsiveness) at each electrode is defined as the electrical current at electrode $i$ multiplied by $\eta$ (4). As the algorithm scales the assumed current to the maximum ECAP amplitude measured in ${{\varvec{M}}}_{{\varvec{o}}}$, *ƞ* can be considered as the synchronized neural response divided by the scaled current. This in turn will depend on several factors including the number of surviving SGNs at electrode $i$, and various attributes of neural tissue such as myelination and survival of the peripheral processes. Because of the scaling, it will also depend on the neural responsiveness and survival elsewhere along the array. Therefore, $\eta$ can be viewed as a measure of ‘synchronized neural responsiveness’ which we refer to simply as ‘neural health’ for brevity. ${\eta }_{i}$ = 0 can therefore be interpreted as an absence of synchronized neural responsiveness at electrode $i$, and ${\eta }_{i}$ = 1 can be viewed as the highest possible synchronized neural responsiveness for that patient with values in between representing relative amounts between these extremes.

The neural health and current spread were modelled by PECAP for 20 hypothetical electrodes beyond the end of the electrode array (10 on either side) to avoid mathematical edge effects unrelated to the intended estimation process that may otherwise occur at both ends of the array. This is also biologically plausible because although PECAP only estimates current spread and neural health patterns for the locations of the active electrodes of the array, these are likely to extend beyond the area covered by the active electrodes, in particular for the most apical cochlear turn (Heutink et al. 2019; Iyer et al. 2018).

## RESULTS: PECAP ALGORITHM VALIDATION

### Computer Simulations

**Fig. 6:**
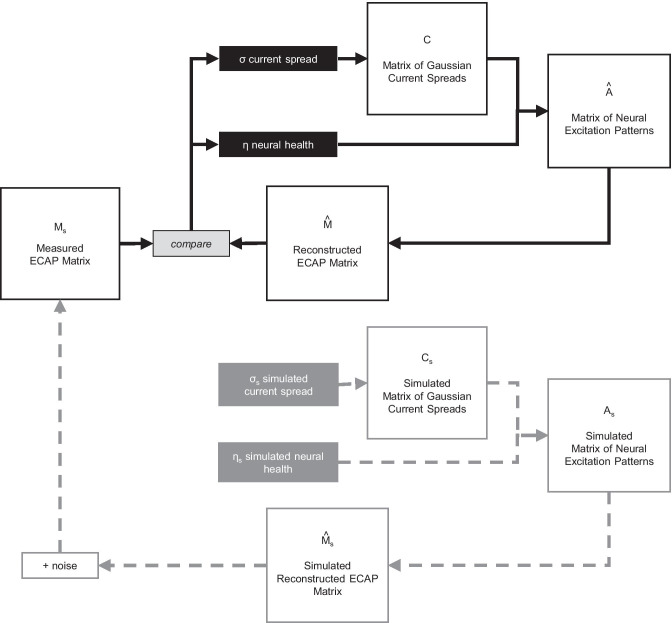
Schematic for the ‘backward model’ of PECAP used for the computational validation of the model accuracy and its sensitivity to noise. The two vectors *σ*_*s*_ and *ƞ*_*s*_ are initialized with pre-defined values, the backwards (grey) part of the algorithm is run to generate a simulated *M*_*s*_ using Eqs. (6), (4), and (3), and random Gaussian noise is added. The algorithm is then run in the normal, forward manner (black) with *σ* and *ƞ* initialized from random numbers as described previously, and the resultant final estimates of *σ* and *ƞ* are evaluated by comparing them to *σ*_*s*_ and *ƞ*_*s*_

The accuracy of PECAP and its robustness to noise was first evaluated using a circular approach based on computer simulations (referred to here as the ‘backward model’ of PECAP), wherein 10 example scenarios of neural health and current spread along the electrode array (*σ*_*s*_ and *ƞ*_*s*_) were artificially generated to simulate ${{\varvec{M}}}_{{\varvec{s}}}$ matrices. The method is depicted in Fig. 6. Because these neural activation patterns were simulated, this method of validation has the advantage that the ground-truth ${{\varvec{A}}}_{{\varvec{s}}}$ matrices are available, which means that in addition to ${\varepsilon }_{{{\varvec{M}}}_{{\varvec{s}}},\widehat{{\varvec{M}}}}$ (which is equivalent to ${\varepsilon }_{{{\varvec{M}}}_{{\varvec{o}}},\widehat{{\varvec{M}}}}$ in Eq. (7) just with ${{\varvec{M}}}_{{\varvec{s}}}$ instead of ${{\varvec{M}}}_{{\varvec{o}}}$), ${\varepsilon }_{{{\varvec{A}}}_{{\varvec{s}}},\widehat{{\varvec{A}}}}$ can also be calculated:

10 $${\varepsilon }_{{{\varvec{A}}}_{{\varvec{s}}},\widehat{{\varvec{A}}}}=\sqrt{\frac{1}{N}\cdot \sum {\left({{A}_{s}}_{p,m}-{\widehat{A}}_{p,m}\right)}^{2}}$$

${\varepsilon }_{{{\varvec{A}}}_{{\varvec{s}}},\widehat{{\varvec{A}}}}$ is a measure of how accurately the PECAP method is able to reconstruct the ground-truth neural activation patterns, whereas ${\varepsilon }_{{{\varvec{M}}}_{{\varvec{o}}},\widehat{{\varvec{M}}}}$ and ${\varepsilon }_{{{\varvec{M}}}_{{\varvec{s}}},\widehat{{\varvec{M}}}}$ indicate how accurately PECAP is able to fit the reconstructed to the measured ECAP data. Only the latter measure (${\varepsilon }_{{{\varvec{M}}}_{{\varvec{o}}},\widehat{{\varvec{M}}}}$) is available when evaluating ECAP data measured with human CI participants (referred to here as the ‘forward model’ of PECAP).

**Fig. 7:**
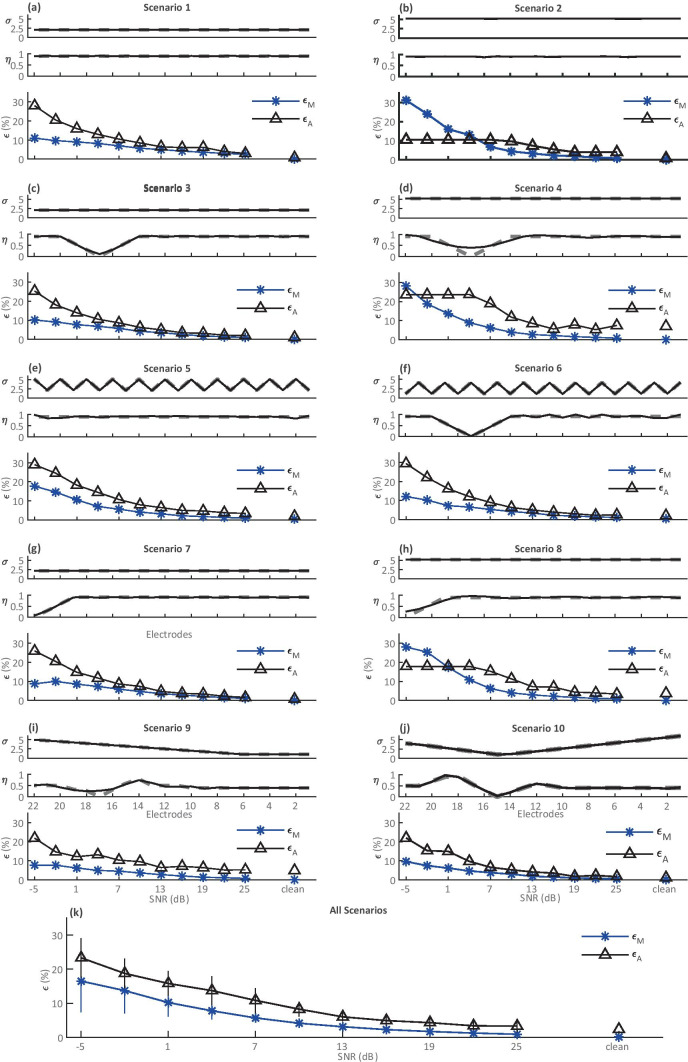
Error (RMSE) profiles as a function of SNR for ten simulated scenarios (**a**–**j**). The neural health vector (*ƞ*) and the current spread vector (*σ*) used to generate the ten scenarios are shown for every electrode (*N* = 22 electrodes) in each plot. In the top two graphs for each simulation scenario, the dashed grey lines indicate the simulated values (*ƞ*_*s*_ and *σ*_*s*_), and the solid black lines indicate the reconstructed predictions of the algorithm (*ƞ* and *σ*) at SNR = ∞ (no noise added). The bottom graph for each simulation scenario shows the ${\varepsilon }_{{{\varvec{M}}}_{{\varvec{s}}},\widehat{{\varvec{M}}}}$ and ${\varepsilon }_{{{\varvec{A}}}_{{\varvec{s}}},\widehat{{\varvec{A}}}}$ values across all SNR levels, normalized by the maximum value in the ${{\varvec{M}}}_{{\varvec{s}}}$ and ${{\varvec{A}}}_{{\varvec{s}}}$ matrices respectively for each condition. Therefore, while the units are indicated as %, the RMSE values have been normalized so they fall on a scale of 0–1. Note that estimates of *ƞ* for the neural dead regions in scenarios 4, 8, and 9 are suboptimal. The across-electrode correlations between original and reconstructed *ƞ* for these scenarios (*ƞ*_*s*_,*ƞ*) are *r*(21) = 0.91, 0.97, and 0.87, respectively (all with *p* < 0.001). The ${\varepsilon }_{{{\varvec{M}}}_{{\varvec{s}}},\widehat{{\varvec{M}}}}$ and ${\varepsilon }_{{{\varvec{A}}}_{{\varvec{s}}},\widehat{{\varvec{A}}}}$ values were then averaged across conditions for all SNRs in the final graph (**k**), in which error bars indicate one standard deviation from the sample mean

**Fig. 8:**
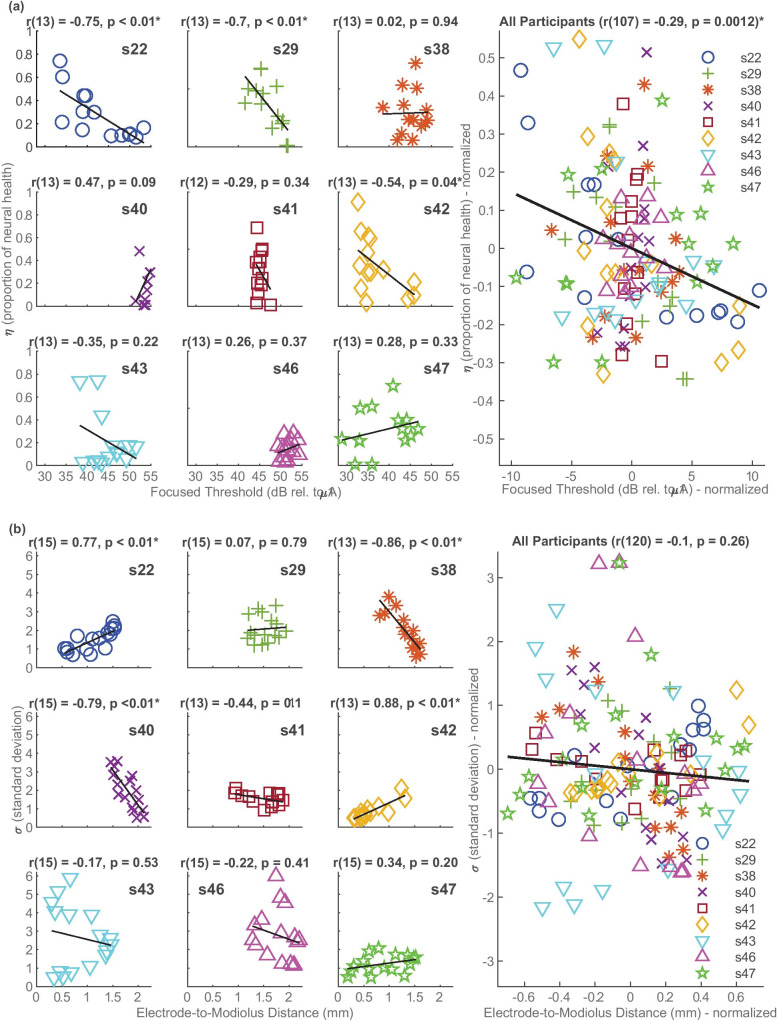
**a** The left-hand plots show the within-participant, across-electrode correlations between Focused Thresholds (obtained using sQP stimulation) and PECAP estimate of neural health (*ƞ*). The right-hand plot shows the combined correlations for all participants, with between-participant differences removed by expressing each value relative to the mean for that participant. **b** This is in the same format as **a** but showing the correlations between electrode-to-modiolus distances and PECAP estimate of current spread (*σ*)

For each of the 10 simulated scenarios, 12 signal-to-noise ratio (SNR) levels were evaluated. Random Gaussian noise was added to the ${{\varvec{M}}}_{{\varvec{s}}}$ matrices before submission to the PECAP algorithm at the following SNR (dB) levels: −5, −2, 1, 4, 7, 10, 13, 16, 19, 22, 25, + ∞. This resulted in a total number of 120 conditions (10 simulated neural activation patterns × 12 SNR values). Each scenario is illustrated by the solid lines in the top two plots in one panel of Fig. 7. Simulation scenarios 1 (Fig. 7a) and 2 (Fig. 7b) both consisted of uniform good neural health and uniform narrow and wide current spread along the electrode array, respectively. Simulation scenarios 3 (Fig. 7c) and 4 (Fig. 7d) emulated 1 and 2 but with the inclusion of a neural dead region towards the apical end of the cochlea centred on electrode 17. Simulation scenarios 5 (Fig. 7e) and 6 (Fig. 7f) consisted of alternating wide and narrow current spread between even and odd electrodes, where 5 had uniformly good neural health and 6 contained a neural dead region, also towards the apical end of the cochlea centred on electrode 17. Simulation scenarios 7 (Fig. 7g) and 8 (Fig. 7h) contained a neural dead region right at the apical edge of the electrode array and uniform narrow and wide current spread, respectively. Finally, simulation scenario 9 (Fig. 7i) had current spread that was wider at the apex and narrower at the base and an uneven pattern of neural health, and simulation scenario 10 (Fig. 7j) had narrow current spread near the middle of the array that got wider towards both ends, also with an uneven pattern of neural health. For all conditions, an arbitrary $\alpha$ value of 150 µV was used to generate the ${{\varvec{M}}}_{{\varvec{s}}}$ matrices, which resulted in a variety of maximum peak ECAP amplitudes in the various simulations. In order to compare the errors between simulations, each error metric (*ε*) was normalized by dividing by the maximum ECAP amplitude in the simulated ${{\varvec{M}}}_{{\varvec{s}}}$ for that simulation and SNR combination. Each panel of Fig. 7 shows the original *ƞ* and *σ* values along the electrode array, the *ƞ* and *σ* reconstructed with an infinitely high SNR (no added noise), and the errors for each simulation (Eqs. 7 and 10) in % as a function of SNR.

The symbols to the right of each plot show the errors obtained for each simulated scenario at infinite SNR (‘clean’, with no noise added). For these ideal conditions, PECAP estimated *σ* with < 2% RMSE for all 10 scenarios, and *ƞ* with < 5% RMSE for 7 of the 10 scenarios. Scenarios 4, 8, and 9 had RMSEs for *ƞ* of 11.19%, 6.96%, and 7.10%, respectively. For these, PECAP was less accurate in estimating areas of low neural health than in the other scenarios, and it should be noted that the neural dead regions in these scenarios were all co-located with areas of relatively wide current spread, illustrating a situation in which the algorithm may have more difficulty. It can also be seen that the algorithm’s RMSE for optimizing ${\varvec{M}}$ is lowest for the most positive SNRs and increases steeply for SNRs below 4–7 dB. The error for the estimation of ${\varvec{A}}$ shows more variability across conditions in its dependence on SNR: on average, it follows a similar pattern to the error for ${\varvec{M}}$, but for some simulations (i.e. 2, 4, and 8) where the current spread is uniformly wide, the RMSE in ${\varvec{A}}$ is constant at low SNRs and drops below 10% or below only for SNRs above 10 dB. It should also be noted that in most conditions, ${\varepsilon }_{{{\varvec{A}}}_{{\varvec{s}}},\widehat{{\varvec{A}}}}$ is greater than ${\varepsilon }_{{{\varvec{M}}}_{{\varvec{o}}},\widehat{{\varvec{M}}}}$. This is unsurprising, as it is ${\varepsilon }_{{{\varvec{M}}}_{{\varvec{o}}},\widehat{{\varvec{M}}}}$ rather than ${\varepsilon }_{{{\varvec{A}}}_{{\varvec{s}}},\widehat{{\varvec{A}}}}$ that is minimized within the PECAP algorithm. An average of ${\varepsilon }_{{{\varvec{M}}}_{{\varvec{o}}},\widehat{{\varvec{M}}}}$ and ${\varepsilon }_{{{\varvec{A}}}_{{\varvec{s}}},\widehat{{\varvec{A}}}}$ across all 10 simulated scenarios is plotted in Fig. 7k, and an evaluation of PECAP’s robustness to noise based on SNR obtained in the real ECAP measurements is described in the fourth algorithm validation section below.

### Association Between Estimated Neural Health and Focused Thresholds in CI Users

It has been suggested that behavioural detection thresholds for focused forms of stimulation should be correlated with neural survival (Goldwyn et al. 2010). DeVries et al. (2016) measured detection thresholds for focused (steered quadrupolar) stimulation (hereafter referred to simply as focused thresholds) on multiple electrodes for nine Advanced Bionics CI users, and also collected ${{\varvec{M}}}_{{\varvec{o}}}$ matrices for those same participants. They kindly provided both sets of data, and the ${{\varvec{M}}}_{{\varvec{o}}}$ matrices were submitted to the PECAP algorithm so as to estimate neural health (*ƞ*) and current spread (*σ*) along the cochlea for each participant. These values were compared to the focused thresholds in the same participants.

Individual participant correlations were performed between the focused thresholds and *ƞ*, with the hypothesis that if *ƞ* accurately models neural health, the two measures should be negatively correlated. To correlate the within-participant, across-electrode variation in *ƞ* and focused thresholds, we performed an ANCOVA with *ƞ* as the dependent variable, focussed threshold as covariate, and participant as a random factor. This analysis is mathematically equivalent to subtracting the mean threshold and *ƞ* across electrodes for each participant from that participant’s scores, and calculating the correlation co-efficient for all data combined. A modest but significant negative correlation was found between *ƞ* and the focused thresholds (*r*(107) = −0.29, *p* = 0.001). Inspection of individual participants’ data showed that 3 of the 9 participants had significant negative correlations (s22: *r*(13) = −0.75, *p* = 0.002, s29: *r*(13) = −0.70, *p* = 0.005, s42: *r*(13) = −0.54, *p* = 0.044), although s42 showed a more moderate correlation than the other two and did not remain significant after Bonferroni corrections for multiple comparisons (for 4 comparative metrics—*ƞ*, *σ*, focused threshold, and electrode-to-modiolus distances (EMD): *p* = 0.05/6 = 0.0083 for 95% significance). It is noted that for the other 6 participants for whom no correlations were found, 5 of them had ${{\varvec{M}}}_{{\varvec{o}}}$ matrices with maximum ECAP amplitudes below 150 µV, suggesting lower SNRs. The maximum ECAP amplitude in the ${{\varvec{M}}}_{{\varvec{o}}}$ matrices of the three participants who showed significant correlations was substantially larger: s22 = 585 µV, s29 = 289 µV, and s42 = 642 µV. It should also be noted that the focused thresholds were obtained using sQP stimulation, whereas the ECAP measurements were obtained using monopolar stimulation at a comfortable level that is expected to be less focused. The different patterns of neural excitation between the two methods of stimulation could have affected the results. The across-electrode correlations are shown for individual participants and for all participants combined in Fig. 8a. For the combined data, the values shown for each participant are normalized to the across-electrode mean for that participant, because the ANCOVA removes between-participant differences (Bland and Altman 1995).

### Association Between Estimated Current Spread and Electrode-to-Modiolus Distances in CI Users

EMD measures were extracted from CT scans in the same participants for whom data was described in the previous section. A detailed description of the collection and processing of these data can be found in DeVries et al. (2016).

We evaluated the within-participant across-electrode correlation between the EMD and *σ*, with the hypothesis that if *σ* accurately models current spread, the two measures should be positively correlated, as an electrode that is farther from the neurons (larger EMD) would be expected to result in a larger current spread. We performed an ANCOVA with *σ* as dependent variable, EMD as co-variate, and participant as a random factor. This analysis did not reveal a significant correlation between EMD and *σ* (*r*(120) = −0.10, *p* = 0.261). At an individual level, 4 of the 9 participants showed significant correlations, 2 of which were positive and 2 of which were negative (s22: *r*(15) = 0.77, *p* < 0.001, s38: *r*(38) = −0.86, *p* < 0.001, s40: *r*(15) = −0.79, *p* < 0.001, s42: *r*(13) = 0.88, *p* < 0.001). The across-electrode correlations are shown for individual participants and for all participants combined in Fig. 8b. For the combined data, the values shown for each participant are normalized to the across-electrode mean for that participant. Possible reasons for the absence of a significant correlation are considered in the ‘Discussion’.

### Detection of Simulated Neural Dead Regions in CI Users

**Fig. 9:**
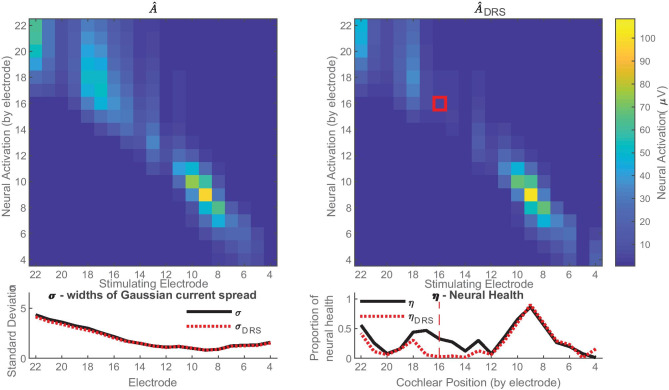
Neural dead region simulation results for participant C03. In the top left, the $\widehat{{\varvec{A}}}$ matrix for neural excitation patterns which PECAP estimates for the standard condition, and in the top right, the ${\widehat{{\varvec{A}}}}_{{\varvec{D}}{\varvec{R}}{\varvec{S}}}$ matrix which PECAP estimates for the neural dead region simulation condition. The neural dead region was simulated for this participant at electrode 16, as indicated by the red box. The graph in the bottom left indicates PECAP estimation of current spread (*σ*) was consistent between the standard condition (black, straight line) and the neural dead region simulation condition (red, dotted line). The graph in the bottom right shows PECAP estimation of neural health (*ƞ*) for both conditions. Poorer estimated neural health is apparent for the neural dead region simulation in comparison to the standard condition from electrodes 13 to 18, but estimates were largely consistent between the two conditions for the other electrodes

The accuracy of the algorithm and its ability to identify neural dead regions was further evaluated using the PECAP data from the seven CI participants for whom neural dead region simulations were performed. It was expected that the occurrence of a simulated neural dead region should not affect the PECAP algorithm’s estimate of current spread (*σ*), but that the estimate of neural health (*ƞ*) would decrease at the location of the electrode used for the dead region simulation while remaining consistent further away from that location. The estimated neural excitation patterns ($\widehat{{\varvec{A}}}$) for one participant (C03) are shown for both the standard and dead region simulation conditions in Fig. 9, along with the *σ* and *ƞ* vectors estimated by PECAP for both conditions. As predicted, the current spread remained consistent between the two conditions (RMSE = 2.27%), and the neural health dropped at and around electrode 16 (the electrode used for the simulation of the dead region). To evaluate whether any reduction in *ƞ* was, as expected, restricted to locations close to the dead-region-simulation electrode, we assumed that the dead region simulation would affect about 5 electrodes, with 2 electrodes on either side of the electrode selected for the dead region simulation. This was based on previous estimates of current spread in CI users, as current levels required to achieve equal loudness have been shown to plateau after the 2nd electrode away from a central one in monopolar mode (Marozeau et al. 2015). For example, in the case of participant C03, it would be expected that the dead region simulation reaches approximately from electrodes 14 to 18 (16 ± 2). For C03, the RMSE between the two condition’s estimated neural health (*ƞ*) for these 5 electrodes was 26.27%. The difference between the *ƞ* vectors for the remaining electrodes across the two conditions was much lower (RMSE = 8.81%), indicating a consistent estimate of neural health for electrodes expected not to be affected by the simulated neural dead region.

**Fig. 10:**
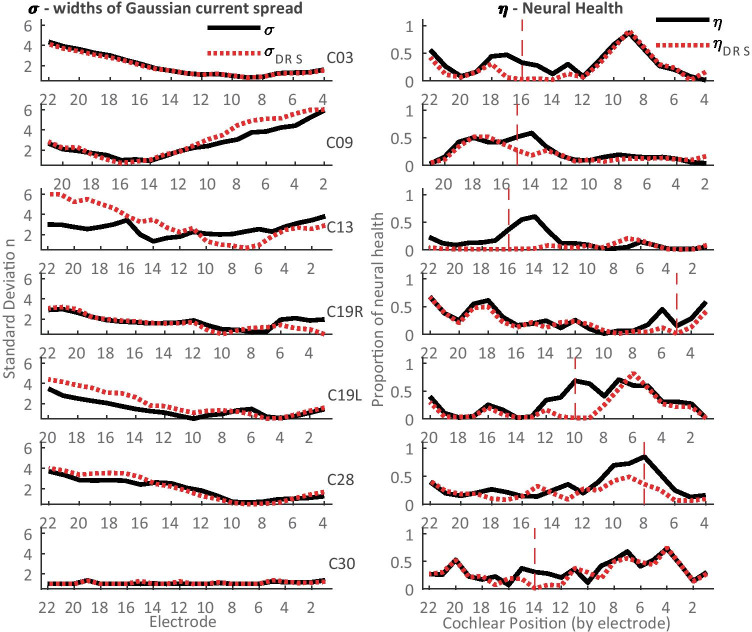
PECAP estimates for *σ* (current spread, left) and *ƞ* (neural health, right) for the seven participants for whom neural dead region simulations were performed. Solid black lines indicate estimations of neural health and current spread from the standard PECAP condition, and dotted red lines indicate estimations in the dead region simulation condition. Vertical dashed red lines in the *ƞ* graphs indicate the electrode that was used for the neural dead region simulation for each participant

The differences in *ƞ* between the two conditions for each participant were not directly comparable, due to the fact that the neural health vector contains proportional values (not absolute values) at each electrode location with respect to the other electrode locations of that CI participant. This was particularly apparent for two participants (C13 and C28) for whom the electrode selected for the neural dead region simulation was at or immediately adjacent to the area with the highest predicted neural health in the standard condition. In order to perform a more direct comparison between the two conditions for these participants in particular, the *ƞ* vector for the dead region simulation dataset was multiplied by the ratio of the maximum ECAP amplitude (in µV) in the ${{\varvec{M}}}_{{\varvec{D}}{\varvec{R}}{\varvec{S}}}$ and the ${{\varvec{M}}}_{{\varvec{o}}}$ matrices for all participants:

11 $${{\eta }^{\mathrm{^{\prime}}}}_{\mathrm{DRS}}=\frac{\mathrm{max}\left({{\varvec{M}}}_{{\varvec{D}}{\varvec{R}}{\varvec{S}}}\right)}{\mathrm{max}\left({{\varvec{M}}}_{{\varvec{o}}}\right)}\cdot {\eta }_{\mathrm{DRS}}$$

where ${{\varvec{M}}}_{{\varvec{o}}}$ is the standard ECAP matrix and ${{\varvec{M}}}_{{\varvec{D}}{\varvec{R}}{\varvec{S}}}$ is the ECAP matrix for the dead region simulation. Figure 10 shows the estimated *σ* and *ƞ* vectors for the standard and neural dead region simulation conditions for all seven participants. It can be seen from visual inspection of the *ƞ* vectors that the PECAP algorithm correctly identified a decrease in the estimated neural health in the expected region for all participants.

As noted above, we would not expect a simulated dead region to affect the estimate of current spread. The first column of Table 2 shows the RMSEs of the *σ* vectors between the two conditions. The highest error occurred for the estimated *σ* vector of C13 (32.58%), which may be due to the low ECAP amplitudes of the neural dead region simulation dataset for this participant (the maximum observed ECAP amplitude in this ${{\varvec{M}}}_{{\varvec{D}}{\varvec{R}}{\varvec{S}}}$ matrix is 45.5 µV). All other RMSEs for *σ* are below 20%, and the overall RMSE is 15.83%, indicating that PECAP estimated current spread with 84% consistency.

**Table 2:** RMSEs for *σ* and *ƞ* for each of the seven participants who participated in the dead region simulation part of the experiment. As expected, errors are consistently higher for *ƞ*_dead_ than for both *σ* and *ƞ*_alive_

Participant	RMSEs (%)			Dead region simulation electrode	Estimated *ƞ* dead region (excluded from *ƞ*alive RMSE)
	*σ*	*ƞ*alive	*ƞ*dead		
C03	2.27%	8.81%	26.27%	16	14–18
C09	13.58%	5.37%	21.82%	15	13–17
C13	32.58%	10.21%	39.50%	16	14–18
C19R	10.21%	6.78%	20.08%	5	3–7
C19L	15.59%	8.91%	44.99%	10	8–12
C28	8.61%	11.74%	31.05%	8	6–10
C30	1.96%	9.39%	20.01%	14	12–16
Across	15.83%	8.97%	30.52%	Total electrodes = 142 (*σ*), 107 (*ƞ*alive), 35 (*ƞ*dead)	

The second column of Table 2 shows that the RMSEs of the *ƞ* vectors for electrodes assumed not to be affected by the dead region simulation (*ƞ*_alive_)—i.e. those located more than 2 electrodes away from the electrode on which the pre-masker pulses were presented—are all below 12%, with an average of 8.97%, indicating approximately 91% consistency. The third column of Table 2 shows the RMSEs of the *ƞ* vectors for electrodes assumed to be affected by the dead region (*ƞ*_dead_). It can be seen from comparing the values in this column with the previous two columns that the RMSEs are higher for *ƞ*_dead_ than for *σ* or *ƞ*_alive_ for all individual participants, as well as when averaged across participants (RMSE = 30.52%). This suggests that PECAP detected the simulated neural dead region in the correct location of the *ƞ* vectors for all participants.

**Fig. 11:**
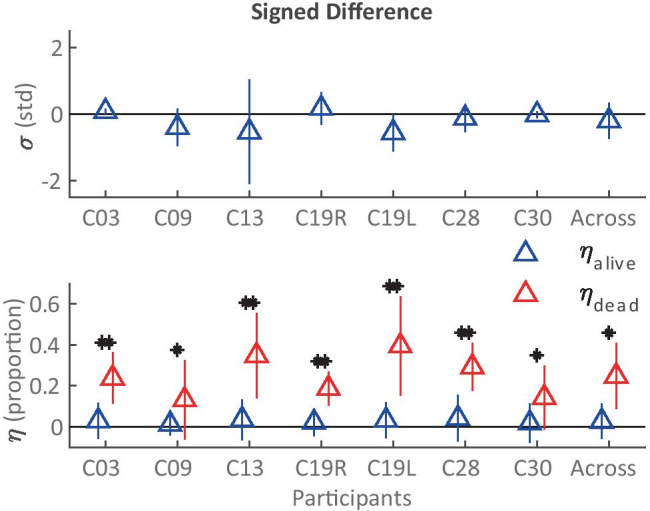
Signed differences for *σ* (current spread) and *ƞ* (neural health) for each of the seven participants who participated in the dead region simulation part of the experiment. The error bars represent one standard deviation from the sample mean. The asterisks show cases where the *ƞ*_alive_ and *ƞ*_alive_ signed differences were statistically significant from each other as a result of a two-tailed *t*-test. Across electrodes, all the individual participants showed *p* < 0.001 and Hedge’s *g*_*s*_ > 0.8 (**) between *ƞ*_alive_ and *ƞ*_alive_ signed differences, except for C09 (*p* = 0.038, Hedge’s *g*_*s*_ = 0.59, df = 19) and C30 (*p* = 0.036, Hedge’s *g*_*s*_ = 0.78, df = 21) (*)

However, as RMSE metrics are calculated with the squared error, they do not inform about the direction of the effect of the dead region simulation on *ƞ*. Therefore, the mean signed differences (MSDs) and their standard deviations were also calculated between the two conditions for each of the metrics in the first three columns of Table 2. It was calculated such that a positive MSD would indicate a reduced value of the metric in the dead region simulation condition. Figure 11 shows these values across electrodes for all individual participants as well as for the across-electrode averages across all participants. For *σ* across participants, MSD = −0.20 (± 0.54 standard deviations). This metric was found not to be statistically significantly than from 0 after a two-tailed *t*-test (*t*(6) = −0.98, *p* = 0.36), indicating no evidence for error bias in estimates of *σ* in one direction or another as a result of the dead region simulation. The estimate of neural health was, across participants, reduced significantly for regions near the simulated dead region (red triangles; MSD for *ƞ*_dead_ = 0.25 (± 0.16)), but not for regions farther away (blue triangles; MSD for *ƞ*_alive_ = 0.026 (± 0.086)). The MSDs for these two populations were significantly different from each other as shown by a two-tailed *t*-test (*t*(6) = 3.26, *p* = 0.017) with a Hedge’s *g*_*s*_ of 1.63, indicating a large effect of the dead-region simulation on *ƞ* across participants in the expected location and direction. All individual participants also showed significant, positive Hedges’ *g* values of above 0.5, and most between 1.4 and 2.1 (above Cohen’s thresholds of 0.5 and 0.8 for a medium and large effect size, respectively (Cohen 1988; Lakens 2013)), indicating that the effect of the dead region simulation observed across participants was also present for each individual participant.

### PECAP’s Robustness to Noise

Finally, we assessed the robustness of PECAP’s neural activation pattern estimates to noise. As the algorithm does not have an internal mechanism for determining the level of confidence it has in its accuracy, it is necessary to develop a way to determine this separately, based on the noise level of any new ${{\varvec{M}}}_{{\varvec{o}}}$ matrix recorded. In the 1st results section (‘Computer Simulations’), it was determined that for all ten simulated scenarios combined, PECAP’s error in replicating $\mathbf{A}$ (${\upvarepsilon }_{{\mathbf{A}}_{\mathbf{s}},\widehat{\mathbf{A}}}$) dropped below 10% for SNRs of 10 dB and above. Therefore, in order to be confident that PECAP’s neural activation patterns are < 10% error, any new ${{\varvec{M}}}_{{\varvec{o}}}$ matrix must have an overall SNR above 10 dB.

**Fig. 12:**
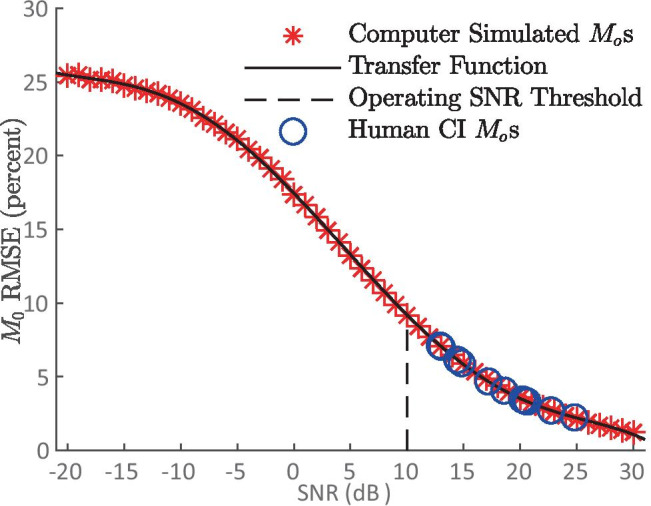
5th order polynomial transfer function (Eq. 12) between RMSEs of ${{\varvec{M}}}_{{\varvec{o}}}$ matrices and SNR values from −20 to 10 dB is indicated as the operating SNR threshold above which PECAP estimates are considered reliable. The 24-sweep RMSEs for the ${{\varvec{M}}}_{{\varvec{o}}}$ s of each of the 11 human CI participants in the second cohort are plotted

However, since SNR cannot be measured directly from a recorded ${{\varvec{M}}}_{{\varvec{o}}}$ matrix, a transfer function must be defined between a measurable property of these data and SNR. The measurable property we selected was the RMSE between repeated measurements of ${{\varvec{M}}}_{{\varvec{o}}}$ using the same stimuli. To address this, 50 new ${{\varvec{M}}}_{{\varvec{o}}}$ matrices for the 10 simulated neural scenarios used in the backwards model of PECAP in ‘Introduction’ were again simulated at each of 51 SNR values ranging from −20 to 30 dB, and 100 RMSE values were then calculated for each condition at each SNR. The average RMSE across simulations was calculated for each SNR (displayed in red asterisks in Fig. 12), and a 5th order polynomial was fit to the data (displayed in the solid black line in Fig. 12):

12 $$f\left(x\right)=-5.70*{10}^{-7}{x}^{5}+ 1.15*{10}^{-5}{x}^{4} + 8.44*{10}^{-4}{x}^{3}-0.012{x}^{2}-0.80x+17.46$$

RMSEs were then calculated for the ${{\varvec{M}}}_{{\varvec{o}}}$ matrices of the CI users. This was done by extracting the first two sets of 12 sweeps from the ECAP waveforms separately from the second two sets of 12 sweeps, and calculating the RMSE between the 2 ${{\varvec{M}}}_{{\varvec{o}}}$ matrices each comprised of 24-sweep ECAP waveforms. These RMSE values were then matched with their corresponding SNR value in the 5th order polynomial fit (displayed in blue circles in Fig. 12). As doubling the number of averages reduces the background noise in the ECAP waveform by a factor of √2 (Undurraga et al. 2012; Stronks et al. 2019) and all previous analyses with PECAP described in this paper were obtained with 48-sweep ECAP waveforms instead of the 24-sweep ${{\varvec{M}}}_{{\varvec{o}}}$ matrices used here, the extracted SNR values (in dB rel. 1 µV) for the human CI data were then increased by 3 dB. This is mathematically equivalent to multiplying the same values in µV on a linear scale by √2. These extrapolated SNR values for each of the human CI datasets from the second participant cohort are included in Table 3. As can be seen from the table, all of these SNR values fall above the 10 dB threshold determined in the 1st algorithm validation section above. This suggests that the PECAP results presented in the previous algorithm validation section with the dead region simulations contained sufficiently low noise levels such that the algorithm can be expected to produce neural activation pattern estimates ($\widehat{\mathbf{A}}$) with > 90% accuracy. We can therefore conclude that the remainder of the human CI data analysed in this study were above the required SNR for PECAP to indicate 90% accuracy in reconstructing neural activation patterns.

**Table 3:** Effective SNRs (in dB) for each of the human CI PECAP ${{\varvec{M}}}_{{\varvec{o}}}$ datasets collected from the participants described in Table 1. It can be seen that all datasets from all participants fall above the 10 dB robustness threshold for PECAP

Participant		C03	C04	C09	C13	C19R	C19L	C25	C26	C28	C29	C30
SNR (dB)	Standard condition	20.19	15.91	21.59	23.62	27.79	23.46	17.46	23.90	25.73	17.83	16.05
	Dead region simulation condition	23.23	n/a	16.04	15.58	28.35	22.98	n/a	n/a	21.64	n/a	29.67

## DISCUSSION

### Choice of Smoothing Constraints

As noted in the ‘Methods: PECAP Algorithm Structure’ section, the inverse problem meant that in order to obtain a unique and robust solution, it was necessary to incorporate smoothness constraints into the PECAP algorithm. Unlike the originl version of the algorithm (Cosentino et al. 2015), the version of PECAP described here used constraints that were biologically plausible. Our method of constraining *ƞ* was informed by a study in anesthetized cats that found that a 2-h exposure to a 12-kHz tone at 124–130 dB SPL caused SGN density to change from approximately 1200 to 300 cells/mm^2^ in the space of 2 mm along the length of the cochlea (Fallon et al. 2020). Assuming that this kind of targeted neural dead region formation is an extreme example of typical variation of neural health in human cochleas, we concluded that for electrodes spaced 0.75 mm apart as in the Cochlear Corporation’s Nucleus Device (Loizou 1998), neural health will not likely vary more than 30% of its overall range between adjacent electrodes (see Eq. 8b).

Our chosen constraints on the current-spread matrix C are consistent with data showing that human cochlear diameters in the first turn are as low as 1.2 mm (Avci et al. 2014). If one reasonably assumes that the electrode array cannot be orthogonal to the ST walls and there is likely to be at least a 45° angle between the array and the wall, there must be a stretch of at least three 0.75-mm-spaced electrodes to reach from one wall of the cochlear tube to the other (1.2 ∗ √2 ≈ 1.7 mm). Assuming that current spread is at its most narrow (i.e. *σ* = 1) when electrodes are closest to the neurons and widest (i.e. *σ* = 6) when they are farthest away, it must take at least three electrodes to span the entire range of possible current-spread values. We therefore assumed that current-spread estimates will not vary more than 50% of their range between adjacent electrodes, and the smoothness constraint was rounded up to 3 from 2.5 (see Eq. 8a) in order to allow the algorithm flexibility regarding this assumption.

In both cases, we performed additional simulations (not shown) using the backward model, which revealed that the predicted patterns of *ƞ* and *σ* were almost identical when the constraints between adjacent electrodes were relaxed from 0.3 to 1 (*ƞ*) and from 3 to 5 (*σ*), respectively.

### Evaluations Using the Backward Model

Using the backward model, it was possible to replicate neural activation patterns (${\varvec{A}}$) with RMSE values of < 10% given simulated ${{\varvec{M}}}_{{\varvec{s}}}$ matrices with SNRs of 10 dB or higher. Assuming that our use of Gaussian noise to simulate measurement noise is reasonable, this indicates that we may expect PECAP’s estimations of neural activation patterns to be at least 90% accurate for an ${{\varvec{M}}}_{{\varvec{o}}}$ matrix that achieves an SNR of 10 dB or higher. The computer simulation validations also revealed three simulated scenarios in which PECAP struggled somewhat to replicate the exact pattern of neural health. In all three of these, PECAP overestimated neural health where a neural dead region was located at the same position along the electrode array as relatively wide current spread. However, it was consistently accurate in its replication of both current-spread and neural-health estimates in all other simulated scenarios. It correctly identified neural dead regions when the current spread was narrow, both in the middle and at the edge of the array. It was also able to reconstruct atypical patterns of alternating current spread with high accuracy, and identified neural dead regions concurrently without increases in error metrics.

### Evaluations Using Behavioural Thresholds and CT-Scan Measures

Recognizing that the backward model of PECAP is a somewhat circular validation, we also took other approaches to evaluate the reliability and accuracy of PECAP’s estimates of current spread and neural health. The data generously provided by DeVries et al. (2016) allowed us to compare PECAP’s estimates of neural health with focused thresholds in a within-participant design. The moderate but significant negative correlation between these two metrics provides evidence in favour of *ƞ* reflecting, at least to some extent, neural health. However, it should be noted that focussed thresholds are not the only measure believed to be affected by neural health (Pfingst et al. 2015). A recent study (Brochier et al. 2021b) obtained several such measures, including the effect of IPG on ECAPs and the effects of pulse rate and stimulus polarity of behavioural thresholds. They found that none of these measures correlated with each other, and used a biophysical model (Joshi et al. 2017) to suggest that different measures reflect different aspects of neural health located at various portions of the auditory nerve. In terms of the PECAP algorithm, neural health is implicitly defined as that aspect of neural health that determines the amplitude of a recorded ECAP, and as previously discussed, therefore represents the synchronous neural responsiveness of the auditory nerve at a given location relative to all other locations along the array for any given patient. It is likely that this definition does not correspond exactly to the aspects of neural health responsible for detection thresholds. For example, a reduction in the number of surviving neurons may affect both behavioural thresholds and ECAPs, whereas a reduction in the synchrony of firing of surviving neurons may affect only the ECAPs.

The DeVries et al. (2016) data also provided the opportunity to compare PECAP’s estimates of current spread with electrode-to-modiolus distances. Although no significant correlation was found here, EMD is also an estimate of expected current spread, not a direct measure. The lack of correlation between the two could therefore be due to inaccuracies in either estimate. It should also be noted that the values from DeVries et al. (2016) were calculated by defining EMD as the distance from the electrode to the medial wall; a recent study showed that ECAP-based metrics were more likely to correlate with EMD when it was calculated from the mid-modiolar axis, as opposed to the medial wall (Schvartz-Leyzac et al. 2020).

### Dead-Region Simulations

We attempted to simulate neural dead regions in human CI users using custom software which enabled the addition of pre-masker pulses prior to each frame of the forward-masking ECAP recording technique. This aimed to simulate localized neural dead regions centred on a chosen electrode by inducing a refractory state in the neurons near that electrode when each ECAP was recorded. PECAP was able to identify differences in estimated neural health located at these simulated dead regions in comparison to when no pre-masker pulses were presented in all participants. Not only this but the fact that it consistently allocated the differences in the neural activation patterns between the two conditions to the neural health estimate *ƞ* rather than to the current spread estimate *σ* indicated that PECAP was able to accurately separate these two aspects of the neural activation patterns. Across all participants and between the two conditions, it achieved 84% consistency in its estimate of current spread, *σ* (the standard deviation of the Gaussian current spread), and 91% consistency in the remainder of the neural health estimate, *ƞ*, demonstrating that the algorithm was also able to give consistent estimates of both attributes when expected. The fact that all participants also showed positive mean signed differences for *ƞ* at the simulated dead region sites indicates that the magnitude of the effect was in the expected direction, and the dead region simulations *reduced* neural health estimates for all individual participants. It should be pointed out, however, that these simulated neural dead regions were likely simulating regions of poor neural health and not regions of complete neural death. In real life, the variation in neural health may be more extreme than can be stimulated in this fashion. Future studies might benefit, for example, from comparing the PECAP algorithm’s neural health estimate between a control group of CI users and a population with a known pattern of neural health such as patients with cochlear nerve deficiency (CND) who display poorer neural survival and responsiveness apically than basally (He et al. 2018). Nevertheless, these results suggest that PECAP is capable of accurately locating regions of reduced neural health in human CI users, and it follows that we can expect it to also identify more extreme changes in neural health such as a truly dead region.

### Estimating the SNR

As there is no internal measure of confidence in the accuracy of PECAP’s estimates of current spread and neural health, we developed a metric of the accuracy of the neural activation patterns for any given ${{\varvec{M}}}_{{\varvec{o}}}$. In order to achieve 90% accuracy in estimating the neural activation patterns (${\varvec{A}}$), we determined that a 10-dB SNR threshold must be achieved by the ${{\varvec{M}}}_{{\varvec{o}}}$. For any future use of PECAP, it is recommended that repeated measures be obtained during data acquisition, such that the SNR can be determined by calculating the RMSE and using the transfer function (Eq. 12). By developing our own ECAP-measurement software so as to separately analyse different subsets of repeats, we were able to do this without increasing the time required to obtain the measurements. If the SNR is above 10 dB, 90% confidence in the neural activation patterns can be expected from PECAP.

### Limitations

We recognize also that there are various limitations of the model. ECAPs only measure the synchronous auditory nerve response and do not capture activity of more central factors which are also understood to contribute to variation in auditory perception between CI users. Therefore, PECAP can only be expected to estimate variation in peripheral auditory factors, and not to capture all variations that impact speech perception outcomes of CI users. ECAPs are also measured at low stimulation rates (i.e. 80 pulses per second), whereas clinical MAPs stimulate at much higher rates. This is a necessary limitation of ECAP measurements but could mean that the PECAP algorithm’s estimates of neural health and current spread are inconsistent with patterns of neural excitation at clinical stimulation rates. ECAPs can also be affected by the characteristics of the recording electrode, which were not considered by the algorithm (Schvartz-Leyzac and Pfingst 2016; Brochier et al. 2021a).

Additionally, in order to be a viable diagnostic tool used in clinical settings, it is desirable to be able to collect PECAP ${{\varvec{M}}}_{{\varvec{o}}}$ matrices in a time-efficient manner. While the algorithm only requires a few seconds on a standard computer to estimate patterns of current spread and neural health once the data is collected, the data acquisition process is currently much slower. It can take up to 45 min to record a PECAP ${{\varvec{M}}}_{{\varvec{o}}}$ matrix using commercially available software in Cochlear Corporation devices, and this does not include the time spent determining current levels for equal loudness across electrodes.

Further work is needed to speed up the data acquisition process as well as transform PECAP into a fully objective tool. This may include evaluating the impact of recording PECAP ${{\varvec{M}}}_{{\varvec{o}}}$ matrices at equal current levels intra-operatively, as well as investigating ways in which the data acquisition time can be reduced without compromising the stability and accuracy of the algorithm’s estimates.

### Clinical Applications

Despite the above-mentioned limitations, the PECAP method could be applied clinically in order to inform additional intervensions as well as augment the clinical decision process during CI fitting today. For example, it is common practice to deactivate an electrode in a CI patient’s MAP when there is evidence that suggests the presence of a neural dead region, such as exceptionally high detection thresholds, impedances, or if the patient provides subjective reports such as ‘poor sound quality’, among other reasons (Sanderson et al. 2019). If PECAP ${{\varvec{M}}}_{{\varvec{o}}}$ matrices were to be collected and analysed as described in this study, the neural health vector (*ƞ*) could be used to objectively inform decisions about which electrodes to deactivate in order to optimize delivery of information from the implant to the neural tissue. Indeed, both the neural health and current spread estimates could be leveraged to inform site-selection strategies to mitigate suboptimal neural excitation patterns. Individual ECAPs are also commonly measured clinically to confirm the responsiveness of neural tissue, and collecting and analyzing PECAP ${{\varvec{M}}}_{{\varvec{o}}}$ matrices could provide a more holistic estimate of the neural activation patterns in these scenarios. While there is potential to use PECAP clinically in these—or other—ways, it will be important to evaluate and confirm the beneficial impact of PECAP-inspired interventions related to CI programming on CI outcomes such as speech perception.

## CONCLUSION

A revised version of the Panoramic ECAP Method (‘PECAP’) was described that estimates patient-specific neural activation patterns in the cochlea in terms of current spread and neural health from electrically evoked compound action potentials in CI users. The algorithm was evaluated using computer simulations, comparisons to other estimates of neural health and current spread, and simulated neural dead regions in human CI users. PECAP reconstructed neural activation patterns with at least 90% accuracy in datasets with SNRs above 10 dB. Moderate correlations with focused thresholds provided evidence in favour of PECAP’s neural health estimate (*ƞ*) to accurately reflect neural health, but also suggested that focused thresholds reflect different (albeit not entirely independent) aspects of auditory neural health than those that determine the amplitude of an ECAP response. The algorithm identified all seven of the simulated regions of reduced neural responsiveness in human CI users, providing further evidence for the accurate estimation of neural health profiles in PECAP’s *ƞ* vector. PECAP could serve as an objective tool to be used in clinical settings to inform and optimize CI programming decisions for individual patients. Further work is required to optimize its data collection time and to evaluate the potential impact of PECAP on CI fitting strategies and their effect on CI speech perception.

## Data Availability

Data will be made available upon request from the corresponding author.

## Code Availability

Code will be made available upon request from the corresponding author.
